# microRNA‐132 is overexpressed in glia in temporal lobe epilepsy and reduces the expression of pro‐epileptogenic factors in human cultured astrocytes

**DOI:** 10.1002/glia.23700

**Published:** 2019-08-13

**Authors:** Anatoly Korotkov, Diede W. M. Broekaart, Leyla Banchaewa, Ben Pustjens, Jackelien van Scheppingen, Jasper J. Anink, Johannes C. Baayen, Sander Idema, Jan A. Gorter, Erwin A. van Vliet, Eleonora Aronica

**Affiliations:** ^1^ Amsterdam UMC, University of Amsterdam Department of (Neuro)Pathology, Amsterdam Neuroscience, Meibergdreef 9 Amsterdam the Netherlands; ^2^ Amsterdam UMC, Vrije Universiteit Amsterdam, Department of Neurosurgery, Amsterdam Neuroscience, De Boelelaan 1117 Amsterdam the Netherlands; ^3^ Swammerdam Institute for Life Sciences, Center for Neuroscience, University of Amsterdam Amsterdam the Netherlands; ^4^ Stichting Epilepsie Instellingen Nederland (SEIN) Heemstede the Netherlands

**Keywords:** epileptogenesis, IL‐1 beta, miRNA, neuroinflammation, TGF‐beta

## Abstract

Temporal lobe epilepsy (TLE) is a chronic neurological disease in humans, which is refractory to pharmacological treatment in about 30% of the patients. Reactive glial cells are thought to play a major role during the development of epilepsy (epileptogenesis) via regulation of brain inflammation and remodeling of the extracellular matrix (ECM). These processes can be regulated by microRNAs (miRs), a class of small non‐coding RNAs, which can control entire gene networks at a post‐transcriptional level. The expression of miRs is known to change dynamically during epileptogenesis. miR‐132 is one of the most commonly upregulated miRs in animal TLE models with important roles shown in neurons. However, the possible role of miR‐132 in glia remains largely unknown. The aim of this study was to characterize the cell‐type specific expression of miR‐132 in the hippocampus of patients with TLE and during epileptogenesis in a rat TLE model. Furthermore, the potential role of miR‐132 was investigated by transfection of human primary cultured astrocytes that were stimulated with the cytokines IL‐1β or TGF‐β1. We showed an increased expression of miR‐132 in the human and rat epileptogenic hippocampus, particularly in glial cells. Transfection of miR‐132 in human primary astrocytes reduced the expression of pro‐epileptogenic COX‐2, IL‐1β, TGF‐β2, CCL2, and MMP3. This suggests that miR‐132, particularly in astrocytes, represents a potential therapeutic target that warrants further in vivo investigation.

## Main Points

- miR‐132 expression is increased in the epileptogenic human and rat hippocampus.
- miR‐132 is expressed by reactive glia.
- miR‐132 attenuates expression of pro-epileptogenic factors in cultured human astrocytes.

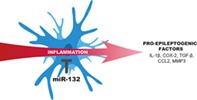

## 1 INTRODUCTION

Epilepsy is a common chronic neurological disease that is estimated to affect more than 65 million people worldwide (WHO [Epilepsy fact sheet], n.d.). Temporal lobe epilepsy (TLE) is the most common form of epilepsy in adults, which is refractory to pharmacological treatment in about 30% of patients. TLE accounts for over 70% of all surgical cases of epilepsy and is often associated with hippocampal sclerosis (HS), tumors, or malformations of cortical development (Blumcke et al., 2017). Neuropathological features associated with TLE include disruption of the blood‐brain barrier (BBB), chronic neuroinflammation, gliosis, and remodeling of the extracellular matrix (ECM; Gorter, van Vliet, & Aronica, 2015). Previous studies in animal TLE models have demonstrated that these pathogenic and adaptive events occur early after an epileptogenic insult in the rodent brain and are present throughout epileptogenesis – the process by which normal brain tissue becomes susceptible to generation of recurrent spontaneous seizures (Loscher, 2011; Pitkanen, Lukasiuk, Dudek, & Staley, 2015). Currently, treatments that prevent or modify epileptogenesis in patients do not exist, thus there is a need for new therapies with anti‐epileptogenic and disease‐modifying properties (Loscher, Klitgaard, Twyman, & Schmidt, 2013).

The neuroinflammatory state of the injured brain is characterized by overexpression of cytokines, chemokines, growth, and complement factors, which are produced by reactive glial cells (van Vliet, Aronica, Vezzani, & Ravizza, 2018; Vezzani, Ravizza, Balosso, & Aronica, 2008). Astrocytes play a key role in this process: under normal physiological conditions, they provide trophic support and maintenance to neurons, but under pathological conditions they undergo morphological and functional changes and acquire reactive properties (Sofroniew & Vinters, 2010). Reactive astrocytes have been shown to display both pro‐inflammatory neurotoxic and anti‐inflammatory neuroprotective phenotypes, which activate various signaling pathways (Anderson et al., 2016; Liddelow et al., 2017; Zamanian et al., 2012). Among the key pathogenic signaling pathways in TLE are the pro‐inflammatory pathway mediated by cytokine interleukin‐1 beta (IL‐1β; Vezzani & Baram, 2007) and the multifunctional transforming growth factor beta (TGF‐β) pathway (Heinemann, Kaufer, & Friedman, 2012). Under pathological conditions these pathways are responsible for the production of the ECM components and remodeling of the ECM structure in the brain (Lau, Cua, Keough, Haylock‐Jacobs, & Yong, 2013), which largely depends on the production and activation of the key proteolytic enzymes of the extracellular space—matrix metalloproteinases (MMPs; Rempe, Hartz, & Bauer, 2016). Chronic neuroinflammation and remodeling of the ECM lead to neurodegeneration and establishment of aberrant synaptic connections, which may ultimately result in epileptogenesis. The modulation of the pathways regulating inflammation and the ECM may be a potential strategy to prevent epileptogenesis.

microRNAs (miRs) is a class of small non‐coding RNAs, which can control entire gene networks at a post‐transcriptional level (Ambros, 2004; Bartel, 2018). miRNAs have been shown to regulate versatile cellular processes both in healthy conditions and in neurological disorders (Cao, Li, & Chan, 2016), including epilepsy (Brennan & Henshall, 2018), which makes them candidate agents for novel anti‐epileptogenic treatments. miRNAs display a dynamic expression profile in the hippocampus of TLE rats and are associated with the regulation of multiple signaling pathways (Gorter et al., 2014). Among the key miRNAs deregulated in epilepsy are miRNAs enriched in astrocytes and associated with brain inflammation (Aronica et al., 2010; Iyer et al., 2012; Korotkov et al., 2018; van Scheppingen et al., 2016; van Scheppingen et al., 2018). In a recent meta‐analysis of differentially expressed miRNAs across rat TLE models, we identified miR‐132 as one of the most commonly upregulated miRNAs (Korotkov, Mills, Gorter, van Vliet, & Aronica, 2017). miR‐132 has been associated with the innate immune response and brain inflammation (Soreq & Wolf, 2011); however, its functions in the brain have been studied mostly in neurons (Aten, Hansen, Hoyt, & Obrietan, 2016). Given the multitude of reactive glial responses in chronic neurological diseases, such as epilepsy, and the inducible nature of miR‐132 expression, we hypothesized that reactive glia may express miR‐132 in TLE. The aim of this study was to characterize the cell‐type specific expression of miR‐132 in human and rat TLE hippocampus and investigate the effects of miR‐132 on the expression of pro‐epileptogenic factors in cultured human astrocytes.

## 2 MATERIALS AND METHODS

### 2.1 Human brain tissue

The cases included in this study were obtained from the archives of the department of Neuropathology of the Amsterdam UMC, the Netherlands. A total of 16 brain specimens were examined from patients who underwent surgery for drug‐resistant TLE. Tissue was obtained and used in accordance with the Declaration of Helsinki and the Amsterdam UMC Research Code provided by the Medical Ethics Committee. All cases were reviewed independently by two neuropathologists and the classification of HS was based on analysis of microscopic examination as described by the International League Against Epilepsy (HS ILAE Type 1, *n* = 12; HS ILAE Type 2, *n* = 4; Blumcke et al., 2013). Control material was obtained during autopsy of people without a history of seizures or other neurological diseases (*n* = 10). Brain tissue was fixed in 10% buffered formalin and embedded in paraffin or directly frozen and stored at ‐80°C. The clinical findings of human samples are presented in Table 1.

**Table 1:** Clinical findings of human samples used for qPCR and in situ hybridization

	Pathology	*n*	Age (years)	Gender (m/f)	Duration of TLE (years)	Age at onset (years)	Number of seizures (per month)
qPCR	Control	14	63 (25–86)	9/5	—	—	—
	TLE–HS	16	39 (24–66)	9/7	20 (5–41)	15 (3–34)	13 (1–36)
ISH	Control	5	48 (31–64)	3/2	—	—	—
	TLE–no HS	5	37 (24–48)	3/2	21 (9–33)	14 (8–18)	38 (2–120)
	TLE–HS	10	35 (24–49)	7/3	20 (5–41)	16 (3–34)	13 (2–32)

*Note*: values are given as mean (minimum–maximum).

Abbreviations: HS, hippocampal sclerosis; TLE, temporal lobe epilepsy.

### 2.2 Experimental animals

Adult male Sprague Dawley rats (Harlan Netherlands, Horst, the Netherlands) were used in this study which was approved by the University Animal Welfare committee and performed in accordance with the guidelines of the European Community Council Directives 2010/63/EU. The rats were housed individually in a controlled environment (21 ± 1°C; humidity 60%; lights on 08:00 a.m. – 8:00 p.m.; food and water available ad libitum).

### 2.3 Electrode implantation and status epilepticus induction

Rats were anesthetized with an intramuscular injection of ketamine (74 mg/kg; Alfasan, Woerden, the Netherlands) and xylazine (11 mg/kg; Bayer AG, Leverkusen, Germany), and placed in a stereotactic frame. In order to record hippocampal EEG, a pair of insulated stainless steel electrodes (70 μm wire diameter, tips 800 μm apart) was implanted into the left dentate gyrus (DG) under electrophysiological control, as described previously (Gorter et al., 2006). A pair of stimulation electrodes was implanted in the angular bundle. Two weeks after recovery from the operation, each rat was transferred to a recording cage (40 × 40 × 80 cm) and connected to a recording and stimulation system (NeuroData Digital Stimulator, Cygnus Technology Inc., Delaware Water Gap, PA) with a shielded multi‐strand cable and electrical swivel (Air Precision, Le Plessis Robinson, France). A week after habituation to the new condition, rats underwent tetanic stimulation (50 Hz) of the hippocampus in the form of a succession of trains of pulses every 13 s. Each train was of 10 s duration and consisted of biphasic pulses (pulse duration 0.5 ms, maximal intensity 700 μA). Stimulation was stopped when the rats displayed sustained forelimb clonus and salivation for several minutes, which usually occurred within 1 hr. Immediately after termination of the stimulation, periodic epileptiform discharges occurred at a frequency of 1–2 Hz which lasted on average 10.0 ± 0.4 hr (status epilepticus, SE). Rats had frequent seizures during this period as observed by both their behavior and EEG.

To determine seizure frequency, continuous EEG recordings (24 hr/day) were made in all rats. Hippocampal EEG signals were amplified (10×) by a high impedance headstage connected to an amplifier (20x; CyberAmp, Axon Instruments, Burlingame, CA), band‐pass filtered (1–60 Hz) and digitized by a computer. A seizure detection program (Harmonie, Stellate Systems, Montreal, Canada) sampled the incoming signal at a frequency of 200 Hz per channel. All EEG recordings were visually screened and seizures were confirmed by trained human observers. Seizures were characterized by synchronized high‐voltage amplitude oscillations and were scored when the amplitude increased more than twofold and lasted for at least 10 s.

### 2.4 Tissue preparation

For in situ hybridization, rats were deeply anesthetized with pentobarbital (Euthasol, AST Farma, Oudenwater, the Netherlands, 60 mg/kg ip) and perfused via the ascending aorta (300 ml 0.37% Na_2_S / 300 ml 4% paraformaldehyde in 0.1 M phosphate buffer, pH 7.4). Rats were perfused at three different time points after SE, each corresponding to the phases of epileptogenesis: the acute phase (1 day post‐SE, *n* = 5), the latent phase (1 week post‐SE, absence of electrographic seizures, *n* = 3) and the chronic phase (3–4 months post‐SE, recurrent spontaneous electrographic seizures are evident, *n* = 7; Gorter, van Vliet, Aronica, & Lopes da Silva, 2001). Control rats (*n* = 4) that were implanted with EEG electrodes, but not stimulated, were also included. The brains were post‐fixated overnight, dissected and paraffin‐embedded. Tissue was sectioned at 6 μm and mounted on pre‐coated glass slides (Star Frost, Waldemar Knittel, Braunschweig, Germany).

For RT‐qPCR analysis, rats were decapitated 1 day after SE (acute phase, *n* = 5), 1 week after SE (latent phase, *n* = 6) or 3–4 months after SE (chronic phase, *n* = 5). Electrode‐implanted control rats were also included (*n* = 5). The brain was dissected and the parahippocampal cortex, which includes mainly the entorhinal cortex and parts of the perirhinal and posterior piriform cortex, was removed by incision at the ventro‐caudal part underneath the rhinal fissure until approximately 5 mm posterior to bregma, as well as the hippocampus. The hippocampus was sliced into smaller parts (200–300 μm) and the DG and Cornu Ammonis (CA1) regions were cut out of the slices in 4°C saline solution under a dissection microscope. All material was frozen on dry ice and stored at −80°C until use.

### 2.5 Cell cultures

Primary fetal astrocyte‐enriched cell cultures were derived from human fetal brain tissue (14–20 weeks of gestation) obtained from medically induced abortions. All material was collected from donors from whom a written informed consent for the use of the material for research purposes was obtained by the Bloemenhove clinic. Tissue was obtained in accordance with the Declaration of Helsinki and the Amsterdam UMC Research Code provided by the Medical Ethics Committee. Tissue samples were collected in astrocyte medium: DMEM/HAM F10 (1:1; Gibco/ThermoFisher Scientific, Waltham, MA), supplemented with 100 units/ml penicillin, 100 μg/ml streptomycin and 10% fetal calf serum (Gibco, Life Technologies, Grand Island, NY). Cell isolation was performed as follows: meninges and blood vessels were removed, tissue was minced and dissociated by incubation with 2.5 mg/ml trypsin at 37°C for 20 min, followed by inactivation of trypsin with astrocyte medium. The tissue was passed through a 70 μm mesh filter and the cell suspension was transferred into a flask with fresh astrocyte medium and maintained in a 5% CO_2_ incubator at 37°C. After 48 hr incubation, the medium was replaced with fresh medium and was subsequently refreshed twice a week. Cultures reached confluence after 2–3 weeks. Astrocytes were used at passages 2–5. More than 98% of the cells in primary culture, as well as in the successive passages were strongly immunoreactive for the astrocytic marker glial fibrillary acid protein (GFAP) and S100β as previously reported (Iyer et al., 2012).

### 2.6 Treatment of cell cultures

Cells were plated in poly‐l‐lysine coated plates (5 × 10^4^ cells/well in 12‐well plates for RNA analysis or 2 × 10^5^ cells/well in 6‐well plates for protein analysis) and were transfected with miR‐132 mimic, antagomir (α‐132) or negative control mimic (mirVana miRNA mimics, Applied Biosystems, Carlsbad, CA). Oligonucleotides were delivered to the cells using Lipofectamine 2000 transfection reagent (Life Technologies, Grand Island, NY) at a final concentration of 50 nM for a total of 24 hr before the stimulation of astrocytes. Astrocytic cultures were stimulated with human recombinant IL‐1β and TGF‐β1 (both 10 ng/ml; Peprotech, Rocky Hill, NJ) for 24 hr (for RNA analysis) or for 48 hr (for protein analysis) before harvesting the cells. Viability of human cell cultures was not influenced by the stimulation, as shown previously (Aronica, Gorter, Rozemuller, Yankaya, & Troost, 2005).

### 2.7 RNA isolation and real‐time quantitative PCR analysis

For RNA isolation, cell cultures, frozen human, or rat brain tissue was homogenized in 700 μl Qiazol Lysis Reagent (Qiagen Benelux, Venlo, the Netherlands). Total RNA, including small RNAs, was isolated using the miRNeasy Mini kit (Qiagen Benelux, Venlo, the Netherlands) according to manufacturer's instructions. The concentration and purity of RNA were determined using a Nanodrop 2000 spectrophotometer (Thermo Fisher Scientific, Wilmington, DE) and the 260/280 ratios of all samples were higher than 2.0 (tissue material) or higher than 1.7 (cell culture material) and did not differ between autopsy control and surgical tissue, indicating that the material was of good quality. To evaluate mRNA expression, oligo dT primers (2.5 nmol) were annealed to 250 ng total RNA (cell culture material) or 2,000 ng (tissue material) in a total volume of 12.5 μl by incubation at 72°C for 10 min, and cooled to 4°C. Reverse transcription was performed by the addition of 12.5 μl RT‐mix, containing: First Strand Buffer (Invitrogen‐Life Technologies), 2 mM dNTPs (Pharmacia, Germany), 30 U RNase inhibitor (Roche Applied Science, Indianapolis, IN) and 400 U M‐MLV reverse transcriptase (Invitrogen ‐ Life Technologies, the Netherlands). The total reaction mix (25 μl) was incubated at 37°C for 60 min, heated to 95°C for 10 min. The cDNA was further diluted with RNase‐free water 3 times (cell culture material) or 10 times (tissue material) and stored at −20°C until use. For each PCR reaction, a master mix contained 1 μl cDNA, 2.5 μl of FastStart Reaction Mix SYBR Green I (Roche Applied Science, Indianapolis, IN), 0.4 μM of both reverse and forward primers. The final volume was adjusted to 5 μl with RNase‐free water. Every PCR reaction was performed in triplicates. A negative control with water instead of cDNA was included in each experiment. For each qPCR *n* = 5 biological replicates (cell culture material) and *n* = 3 technical replicates were used. Technical replicates were excluded if the value of a replicate was more than 10% different from the average of the replicates. The cycling conditions were carried out as follows: initial denaturation at 95°C for 5 min, followed by 45 cycles of denaturation at 95°C for 15 s, annealing at 65°C for 5 s and extension at 72°C for 10 s. The fluorescent product was measured by a single acquisition mode at 72°C after each cycle. The primers used for the study are listed in Table S1. The geometric mean of elongation factor 1α (EF1α) and chromosome 1 open reading frame 43 (C1orf43) expression was used for normalization.

The expression of miR‐132‐3p was analyzed using Taqman microRNA assays (Assay No. 000457; Applied Biosystems, Foster City, CA) according to the manufacturer's instructions. U6B small nuclear RNA (RNU6B; Assay No. 001093; Applied Biosystems) was used for normalization of miRNA expression in brain tissue and U6 snRNA (Assay No. 001973; Applied Biosystems) was used for normalization in culture samples. cDNA was generated using Taqman MicroRNA reverse transcription kit (Applied Biosystems, Foster City, CA, USA) according to the manufacturer's instructions. The PCRs were run on the Roche LightCycler 480 (Roche Applied Science, Basel, Switzerland) with a 384‐multiwell format.

Quantification of data was performed using LinRegPCR in which a baseline correction and window‐of‐linearity were determined for each sample separately, followed by a linear regression analysis on the Log (fluorescence) per cycle to fit a straight line through the PCR data set. The slope of this line is used to determine the PCR efficiency of each individual sample. The mean PCR efficiency per amplicon and the Ct value per sample are used to calculate a starting concentration N0 per sample, which is expressed in arbitrary fluorescence units (Ruijter et al., 2009). The starting concentration N0 of each specific product was then divided by the geometric mean of the starting concentrations N0 of the reference genes and this ratio was compared between groups.

### 2.8 Western blot analysis

Cells were harvested at 48 hr after treatment. The cells were washed with ice‐cold PBS and homogenized in ice‐cold lysis buffer (50 mM Tris–HCl pH 7.4, 150 mM of NaCl, 1% NP‐40, 0.5% sodium deoxycholate) supplemented with protease inhibitor (EDTA‐free protease mixture inhibitor and phosphatase inhibitor (Roche Diagnostics, Almere, The Netherlands) by incubating on ice for 10 min and collected using a cell scraper. The homogenates were centrifuged at 12,000xg for 10 min and the supernatant was used for further analysis. Protein content was determined using the bicinchoninic acid method (Smith et al., 1985). For electrophoresis, equal amounts of proteins (20 μg/lane for culture samples) were separated using sodium dodecyl sulfate polyacrylamide gel electrophoresis on a 10% gel. Subsequently, separated proteins were transferred onto polyvinylidene difluoride membranes (Immobilon‐P; Merck, Darmstadt, Germany) for 90 min at 100 V, using a wet electroblotting system (BioRad, Hercules, CA). Blots were blocked for 1 hr in 5% non‐fat dry milk in Tris‐buffered saline‐Tween (TBS‐T; 20 mM Tris, 150 mM NaCl, 0.1% Tween 20, pH 7.5). Blots were incubated overnight at 4°C with primary antibodies: anti‐TGF‐β2 (1:500 rabbit polyclonal, sc‐90, Santa Cruz Biotechnology, Santa Cruz, CA), anti‐COX‐2 (1:1,000, D5H5 rabbit monoclonal, Cell Signaling Technology, Leiden, the Netherlands), anti‐β‐actin (1:30,000, mouse monoclonal, clone C4, Merck, Darmstadt, Germany) or anti‐β‐tubulin (1:10,000, mouse monoclonal, Sigma‐Aldrich, St. Louis, MO). After several washes in 5% non‐fat dry milk in TBS‐T, blots were incubated with secondary antibodies polyclonal goat anti‐rabbit immunoglobulin‐HRP (1:2,500, Dako, Glostrup, Denmark) or goat anti‐mouse immunoglobulin‐HRP (1:2,500, Dako, Glostrup, Denmark) for 1 h. After several washes in TBS‐T, immunoreactivity was visualized using ECL PLUS Western blotting detection reagent (GE Healthcare Europe, Diegen, Belgium). Expression of β‐actin or β‐tubulin was used as loading control. Chemiluminescent signal was detected using ImageQuant LAS 4000 analyzer (GE Healthcare, Eindhoven, the Netherlands). Precision Plus Protein Dual Color Standards (Bio‐Rad, Richmond, CA) was used to determine the molecular weight of the proteins. For the quantitative analysis of the blots the band intensities were measured densitometrically using ImageJ software (U.S. National Institutes of Health, Bethesda, MD).

### 2.9 In situ hybridization on human and rat brain tissue

Paraffin‐embedded brain tissue was deparaffinised in xylene and rinsed in ethanol (2× 100%, 1× 70%) and sterile water. Antigen retrieval was performed using a pressure cooker in sodium citrate buffer, pH 6.0, at 121°C for 10 min. The oligonucleotide probe against miR‐132‐3p (Table S1) contained LNA modifications, 2‐O‐methyl modifications and a double digoxygenin (DIG) label (RiboTask ApS, Odense, Denmark). Sections were incubated with the probe (33 nM) in hybridization mix (600 mM NaCl, 10 mM HEPES, 1 mM EDTA, 5× Denhardts, 50% formamide) for 1 hr at 56°C. Sections were washed with 2× saline‐sodium citrate buffer for 2 min, 0.5× for 2 min, 0.2× for 1 min (in agitation). After washing with sterile PBS, sections were blocked for 15 min with 1% BSA, 0.02% Tween 20 and 1% normal goat serum. Hybridization was detected with sheep alkaline phosphatase (AP)‐labeled anti‐DIG antibody (1:1,500, Roche Applied Science, Basel, Switzerland). Nitro‐blue tetrazolium chloride (NBT)/5‐bromo‐4‐chloro‐3′‐indolyphosphate p‐toluidine salt (BCIP) was used as chromogenic substrate for AP (1:50 diluted in NTM‐T buffer: 100 mM Tris, pH 9.5; 100 mM NaCl; 50 mM MgCl_2_; 0.05% Tween 20). Negative control assays were performed without the probe (sections were blank). For in situ hybridization with double labelling, the sections were first incubated in 0.3% H_2_O_2_/methanol solution for 20 min to block endogenous peroxidase activity, followed by in situ hybridization and then by immunohistochemistry. Slides were washed with PBS and incubated for 1 hr at room temperature with primary antibodies on normal antibody diluent (Klinipath, Olen, Belgium): mouse anti‐GFAP (1:4,000, Sigma‐Aldrich, St. Louis, MO), mouse anti‐NeuN (1:2,000, MAB377, Chemicon, Temecula, CA), mouse anti‐CD34 (1:600, Immunotech Laboratories, Monrovia, CA), mouse anti‐HLA‐DR/DP/DQ (1:100, clone CR3/43 Agilent, Santa Clara, CA), rabbit anti‐Iba1 (1:2,000, Wako Chemicals, Neuss, Germany), mouse anti‐vimentin (1:1000, clone V9, Dako, Glostrup, Denmark), mouse anti‐EAAT1 (1:100, clone 10D4, Monosan, Uden, the Netherlands), rabbit anti‐GS (1:100, G2781, Sigma‐Aldrich, St. Louis, MO), or rabbit anti‐STAT3 (1:100, DIA5 XP, Cell Signaling, MA). After washing with PBS, sections were stained with a polymer‐based horseradish peroxidase (HRP) immunohistochemistry detection kit (Brightvision plus kit, ImmunoLogic, Duiven, The Netherlands) according to the manufacturer's instructions. The visualization of the antibody–antigen binding was done using 3‐amino‐9‐ethylcarbazole (AEC; Sigma‐Aldrich, St. Louis, MO), which in the presence of hydrogen peroxide undergoes chromogenic oxidation catalyzed by HRP with the formation of a red precipitate.

### 2.10 Evaluation of in situ hybridization

The expression of miR‐132 was analyzed in the hippocampus of human and rat brain tissue using two different approaches: the optical density (OD) approach and the in situ reactivity score (IRS) approach. For the OD approach, the OD above threshold of the hybridization signal in microphotographs obtained by in situ hybridization was measured using ImageJ. Measurements were performed in the granule cell layer (GCL), molecular layer, and hilus of the DG, the pyramidal cell layer (PCL) of CA1, as well as the stratum radiatum and stratum oriens. For the in situ reactivity approach, the intensity of the hybridization signal was evaluated in neurons and glia in the DG, hilus, and CA1 using a scale of 1–4 (1: no; 2: weak; 3: moderate; 4: strong hybridization signal). The score represents the predominant signal intensity found in each case. Furthermore, the relative number of positive cells (0: no; 1: single to 10%; 2:11–50%; 3: >50%) was also evaluated in these areas. Then the in situ reactivity score (IRS) was calculated by multiplying the intensity score by the relative number score as described previously (Broekaart et al., 2017).

### 2.11 Statistical analysis

Statistical analyses were performed using IBM SPSS Statistics 21. Comparisons between groups were done using the Mann–Whitney *U* test. A value of *p* < .05 was assumed to indicate significant difference.

## 3 RESULTS

### 3.1 Higher miR‐132 expression in the rat epileptogenic hippocampus

**Figure 1:**
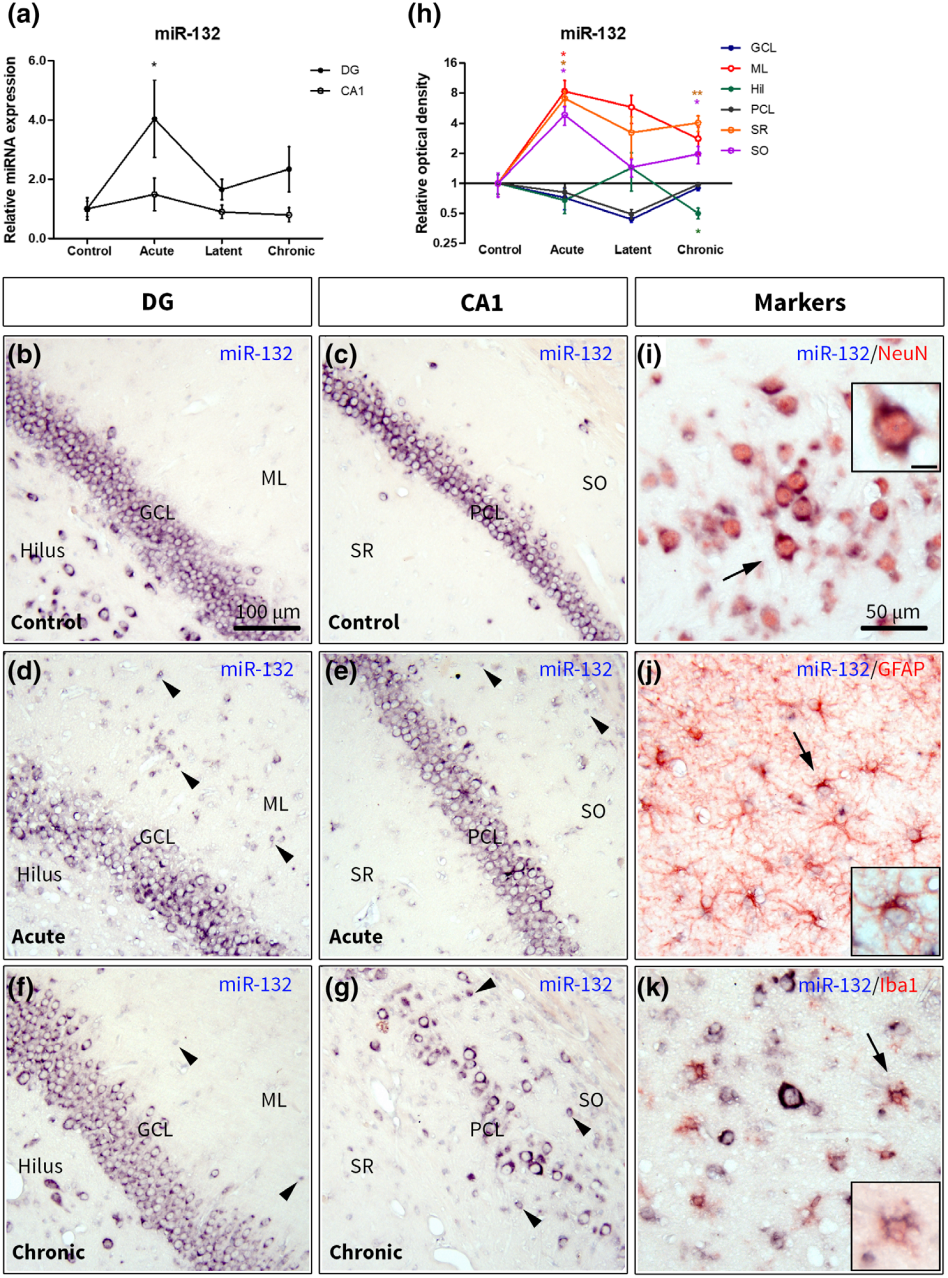
Expression of miR‐132 in the rat hippocampus. (a)—Taqman RT‐qPCR showed a 2.1‐fold higher (*p* < .05) miR‐132 expression in the DG during the acute stage; (b–g)—in situ hybridization for miR‐132 in the control (b, c), during the acute (d, e) and the chronic (f, g) stages. Mostly neuronal expression was observed in the control DG (b) and CA1 (c). Higher expression was observed in cells with glial morphology in the DG ML (d, black arrowheads), CA1 SO and CA1 SR (e) during the acute stage as well as during the chronic stage (f, g); (h) OD analysis in different hippocampal layers relative to control; (i–k)—double labeling of miR‐132 with cell‐type specific markers: miR‐132 was co‐localized with NeuN (i), GFAP (j) and Iba1 (k), the insets show higher magnification of cells indicated by black arrows; scale bar in (b–g) 100 μm; scale bar in (i–k) 50 μm; scale bar in (i) inset 10 μm and applies to (j, k) insets; DG, dentate gyrus; GCL, granule cell layer; Hil, hilus; ML, molecular layer of the DG; PCL, pyramidal cell layer; SR, stratum radiatum; SO, stratum oriens; Mann–Whitney *U* test *p < .05, **p < .01 [Color figure can be viewed at https://wileyonlinelibrary.com]

RT‐qPCR analysis showed that the expression of miR‐132 was 2.1‐fold higher (*p* < .05) in the dentate gyrus (DG) during the acute stage (1 day post‐SE) of epileptogenesis as compared to control; however, not during the latent stage (1 week post‐SE) or the chronic stage (3–4 months post‐SE; Figure 1a). The expression of miR‐132 in CA1 did not differ from control at any time point (Figure 1a). We further assessed the expression and distribution of miR‐132 using in situ hybridization. The expression of miR‐132 throughout the control hippocampus was predominantly observed in neurons, including DG granule cells and hilar cells (Figure 1b), as well as pyramidal cells of CA1‐4 (Figure 1c). The pattern of miR‐132 expression was different during the acute stage, with a stronger hybridization signal observed in individual neurons in the DG and CA1, as well as a higher number of cells with glial morphology in the DG molecular layer (ML; Figure 1d), CA1 stratum radiatum (SR), and CA1 stratum oriens (SO; Figure 1e), which were not observed in the control hippocampus. The OD analysis demonstrated that the expression of miR‐132 during the acute stage was ~5‐8‐fold higher in the DG ML, CA1 SR, and CA1 SO as compared to control (Figure 1h, all *p* < .05). The IRS approach, in which neurons were discriminated from glial cells, showed that neuronal expression of miR‐132 was higher in the DG, CA1, and hilus at the acute stage (Table 2, *p* < .05) as compared to control. Higher expression at the acute stage was also observed in the cells with glial morphology in the DG (*p* < .05), in the hilus (*p* < .01), and in the CA1 (*p* < .01; Table 2). During the latent stage, the OD did not differ from control in any of the analyzed hippocampal layers (Figure 1h); however, a higher expression in the cells with glial morphology was observed in the CA1 (Table 2, *p* < .05). During the chronic stage the number of stained cells with glial morphology in the DG ML was less pronounced (Figure 1f) as compared to the latent stage; however, numerous stained cells were observed in CA1 SO and SR (Figure 1g). The OD during the chronic stage was ~2‐4‐fold higher in the SR (*p* < .01) and SO (*p* < .05), while twofold lower in hilus (*p* < .05) as compared to control (Figure 1h). Higher expression at the chronic stage was also observed in the cells with glial morphology in the DG (*p* < .05), hilus (*p* < .05), and CA1 (all *p* < .01; Table 2). The OD did not change in the GCL and PCL at any time point as compared to control (Figure 1h). Double labeling of miR‐132 with cell‐type specific markers revealed that the expression of miR‐132 was co‐localized with the neuronal marker NeuN (Figure 1i), the astrocyte marker GFAP (Figure 1j), and the microglia marker Iba1 (Figure 1k).

**Table 2:** In situ reactivity scores for miR‐132^+^ cells in the rat hippocampus

	DG		Hilus		CA1	
	Neurons	Glia	Neuron	Glia	Neurons	Glia
Control	4.5 (3–9)	0.5 (0–2)	4.5 (3–9)	0	6 (3–9)	0
Acute	9 (9–12)*	9 (1–12)*	12 (9–12)*	9 (9–12)**	12 (9–12)*	9 (6–12)**
Latent	6 (6–9)	0	6		6 (6–9)	9*
Chronic	6 (3–9)	3 (1–6)*	6 (3–9)	6 (0–6)*	6 (3–12)	4 (1–6)**

*Note*: The expression is defined as intensity score (1: no; 2: weak; 3: moderate; 4: strong hybridization signal) multiplied by the relative number of positive cells score (0: no; 1: single to 10%; 2: 11–50%; 3: >50%); miR‐132 in situ reactivity scores are given as median (minimum–maximum); DG, dentate gyrus; Mann–Whitney *U* test, *p < .05: **p < .01.

### 3.2 Higher miR‐132 expression in the human hippocampus

**Figure 2:**
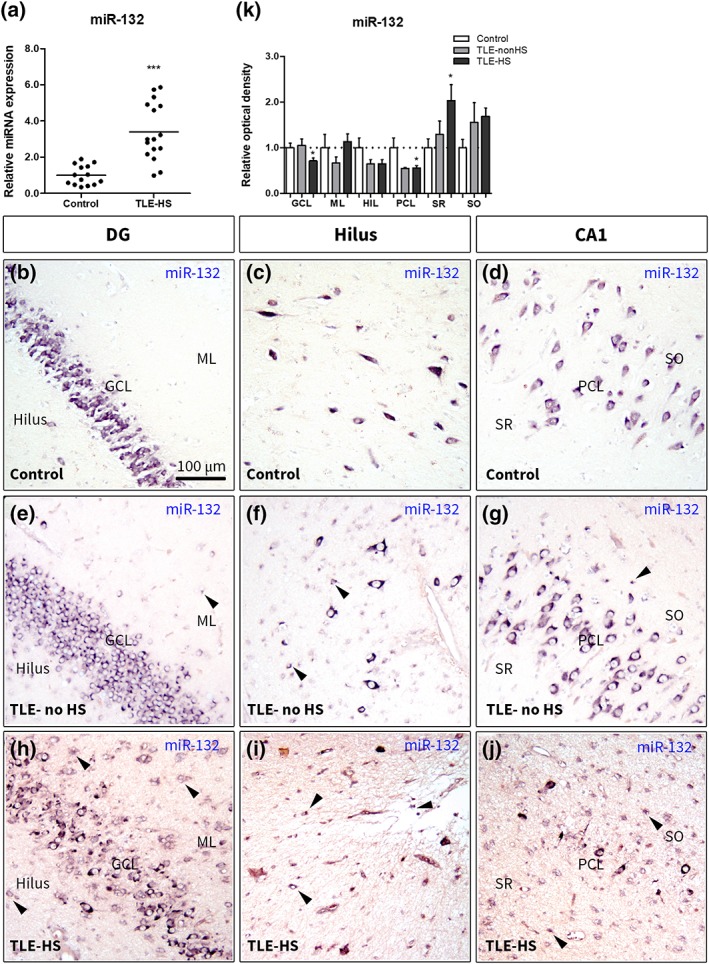
Expression of miR‐132 in the human hippocampus. (a)—Taqman RT‐qPCR showed a 3.4‐fold higher (*p* < .001) miR‐132 expression in the hippocampus of patients with TLE‐HS; (b–j)—in situ hybridization for miR‐132 in the hippocampus of TLE without HS (e–g) and TLE with HS (h–j) patients compared to autoptic control (b–d); mostly neuronal expression of miR‐132 was observed in the DG (b), hilus (c), and CA1 regions of control hippocampus. Higher expression was observed in cells with glial morphology in the DG ML (e, here and further indicated by black arrowheads), hilus (f) and CA1 (g) which was most prominent in the DG ML of TLE‐HS (h); hilus (i) and CA1 (j) and was associated with GCL dispersion and severe neuronal loss in the hilus and CA1 PCL; (k)—OD analysis showed a higher expression in CA1 SR (*p* < .05) and a lower expression in the GCL and PCL (both *p* < .05) of the TLE‐HS hippocampus relative to control; DG, dentate gyrus; GCL, granule cell layer; Hil, hilus; ML, molecular layer of the dentate gyrus; PCL, pyramidal cell layer; SO, stratum oriens; SR, stratum radiatum; scale bar 100 μm; Mann–Whitney *U* test *p < .05, ***p < .001 [Color figure can be viewed at https://wileyonlinelibrary.com]

Following the findings in the rat hippocampus, we investigated the expression of miR‐132 in resected hippocampi from patients with drug‐resistant TLE as well as from autopsy controls (Table 1). RT‐qPCR analysis showed a 3.4‐fold higher expression of miR‐132 (*p* < .001) in patients with TLE and hippocampal sclerosis (TLE‐HS) as compared to controls (Figure 2a). In situ hybridization demonstrated that the pattern of miR‐132 expression and distribution in control human hippocampus was similar to the one observed in control rat hippocampus with the hybridization signal detected predominantly in neurons of the principal cell layers and hilus (Figure 2b–d). The expression of miR‐132 was higher in the DG, hilus, and CA1 regions of patients with TLE (Figure 2e–j) and was most pronounced in patients with TLE‐HS (Figure 2h–j). OD analysis showed a 1.4‐fold lower expression of miR‐132 in the DG GCL (*p* < .05) and 1.8‐fold lower expression in CA1 PCL (p < .05), but two‐fold higher expression in the CA1 SR (*p* < .05) as compared to control. An upward trend was also observed in SO (*p* = .053; Figure 2k). The analysis of individually stained cells by the IRS approach revealed a higher miR‐132 expression in the cells with glial morphology in the DG in both TLE without HS (TLE‐no HS; *p* < .05) and TLE‐HS (*p* < .01), in the hilus in TLE‐no HS (*p* < .05) and TLE‐HS (*p* < .01), as well as in the CA1 in TLE‐no HS (*p* < .01) and TLE‐HS (*p* < .01) as compared to control (Table 3).

**Table 3:** In situ reactivity scores for miR‐132^+^ cells in the human hippocampus

	DG		Hilus		CA1	
	Neurons	Glia	Neurons	Glia	Neurons	Glia
Control	6 (3–6)	1 (0–2)	7.5 (3–9)	0	6 (3–9)	0
TLE–no HS	9 (6–12)	9 (1–12)*	12 (6–12)	9 (1–12)*	12 (6–12)	9 (1–12)**
TLE–HS	12 (3–12)*	12 (6–12)**	12 (6–12)**	11 (6–12)**	12 (6–12)*	12 (6–12)**

*Note*: The expression is defined as intensity score (1: no; 2: weak; 3: moderate; 4: strong hybridization signal) multiplied by the relative number of positive cells score (0: no; 1: single to 10%; 2:11–50%; 3: >50%); miR‐132 in situ reactivity scores are given as median (minimum‐maximum); DG, dentate gyrus; Mann–Whitney *U* test, *p < .05: **p < .01.

**Figure 3:**
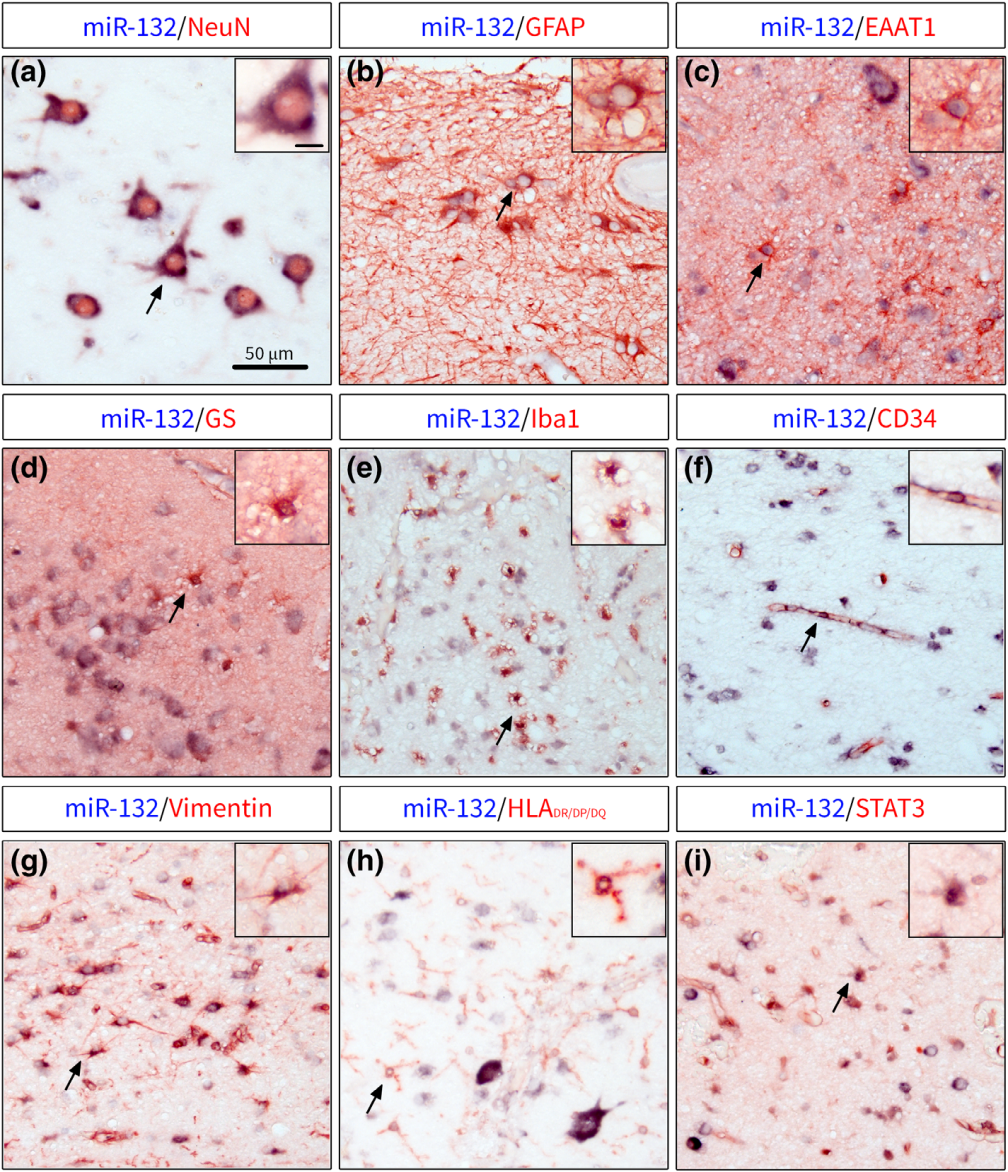
Double labeling of miR‐132 with cell type‐specific markers in human TLE‐HS. (a)—miR‐132 was expressed in neurons throughout hippocampus, including granule cells, pyramidal cells and hilar neurons; co‐localization of miR‐132 was also found with markers of astrocytes GFAP (b), EAAT1 (c) and GS (d); miR‐132 was also co‐localized with Iba1^+^ microglial cells (e) in the areas of glial scar in CA1 and with CD34 (f) associated with blood vessels; the co‐localization with reactivity markers showed miR‐132 expression in vimentin^+^ cells with astrocytic morphology (g), HLA‐DR/DP/DQ‐expressing cells with microglial morphology (h) and STAT3^+^ cells in the CA1; black arrows indicate cells shown in higher magnification in insets; scale bar 50 μm; scale bar in inset a =10 μm and applies for insets a‐i [Color figure can be viewed at https://wileyonlinelibrary.com]

Double labeling of miR‐132 with cell‐type specific markers showed that miR‐132 co‐localized with virtually all NeuN^+^ cells in the human TLE‐HS hippocampus, including granule cells, pyramidal cells and neurons in the hilus (Figure 3a). In addition to neuronal expression, miR‐132 was found in astrocytes based on co‐localization with GFAP (Figure 3b), excitatory amino acid transporter 1 (EAAT1; Figure 3c) and glutamine synthetase (GS; Figure 3d). The expression in astrocytes was found throughout hippocampus, in the CA1 and hilus, as well as in the DG ML. The co‐localization of miR‐132 with the marker of microglial cells Iba1 (Figure 3e) was also observed, which was most prominent in the CA1. Additionally, miR‐132 expression was closely associated with the brain endothelial cell marker CD34 (Figure 3f). We further assessed whether miR‐132 expression was co‐localized with markers of astrocyte and microglia reactivity. Co‐localization of miR‐132 was found with vimentin (Figure 3g), as well as a marker of reactive microglia—human leukocyte antigen (HLA‐DR/DP/DQ; Figure 3h) and signal transducer and activator of transcription 3 (STAT3) throughout the hippocampus. The co‐localization with these markers was especially prominent in the areas of extensive gliosis and around lesions in the CA1 layer. Thus, we found that in the human TLE‐HS hippocampus miR‐132 was localized not only to neuronal cells, as previously reported, but also non‐neuronal cells, especially reactive glia.

### 3.3 miR‐132 overexpression in astrocytes modulates TGF‐β pathway

**Figure 4:**
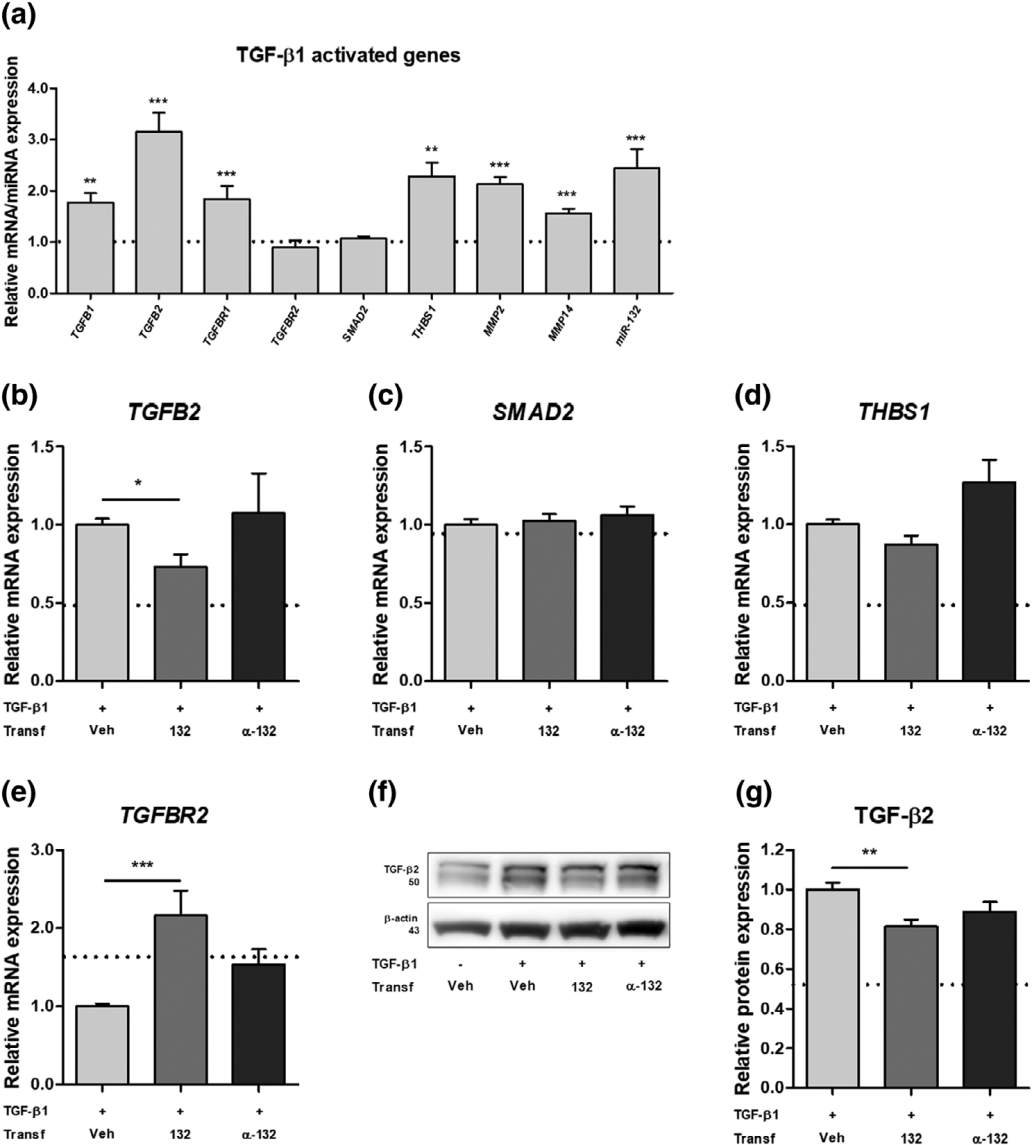
miR‐132 overexpression modulates TGF‐β pathway. The stimulation of primary human fetal astrocytes with TGF‐β1 resulted in higher expression of *TGFB1* (*p* < .01), *TGFB2* (*p* < .001), *TGFBR1* (*p* < .001), *THBS1* (*p* < .01), *MMP2* (*p* < .001), *MMP14* (*p* < .001), and miR‐132 (*p* < .001) as compared to non‐stimulated control (a); the higher expression of *TGFB2* was attenuated by miR‐132 mimic transfection (*p* < .05) after TGF‐β1 stimulation (b); no change was found in expression of *SMAD2* (c) and *THBS1* (d); the expression of *TGFBR2* was higher after miR‐132 transfection (*p* < .001) (e); western blot analysis showed that the expression of TGF‐β2 protein was lower (*p* < .01) after miR‐132 transfection (f, g); the data was normalized to the stimulated control condition and the dotted line indicates non‐stimulated control; veh, vehicle (Lipofectamine 2000); α‐132 indicates antagomir; *n* = 5 for RT‐qPCR, *n* = 3 for western blot, Mann–Whitney *U* test, *p < .05, **p < .01, ***p < .001

We further investigated the potential role of miR‐132 in primary cultures of human fetal astrocytes. Pathway enrichment analysis of miR‐132 target genes using the Diana mirPath v.3 software (Vlachos et al., 2015) revealed TGF‐β as the most enriched pathway regulated by miR‐132. Stimulation of astrocytes with TGF‐β1 resulted in higher expression of the genes involved in TGF‐β signaling, such as transforming growth factor beta 1 (*TGFB1*; 1.8‐fold, *p* < .01) and 2 (*TGFB2*; 3.one‐fold, *p* < .001), the TGFB receptor 1 *TGFBR1* (1.8‐fold, *p* < .001) and thrombospondin 1 (*THBS1*; 2.3‐fold, *p* < .01), but had no effect on the expression of *TGFBR2* and SMAD family member 2 (*SMAD2*; Figure 4a). Higher expression of matrix metalloproteinase genes *MMP2* (2.1‐fold, *p* < .001) and *MMP14* (1.6‐fold, *p* < .001) was also found after TGF‐β1 stimulation (Figure 4a). Moreover, endogenous expression of miR‐132 was also 2.4‐fold higher (*p* < .001) after TGF‐β1 stimulation (Figure 4a). We found that the 3’ untranslated region (UTR) of *TGFB2* mRNA was a target of miR‐132 as predicted by the Targetscan software (Agarwal, Bell, Nam, & Bartel, 2015; Figure S1a). Exogenous overexpression of miR‐132 by mimic oligonucleotides led to a 30% lower expression of *TGFB2* (*p* < .05) under TGF‐β1 stimulation (Figure 4b). The expression of other predicted miR‐132 target genes *SMAD2* (Figure 4c) and *THBS1* (Figure 4d) did not change following miR‐132 modulation. Overexpression of miR‐132 also resulted in 2.2‐fold higher expression of *TGFBR2* (*p* < .05, Figure 4e). Western blot analysis for TGF‐β2 protein confirmed a 20% lower expression after miR‐132 overexpression (Figure 4f, g). These data shows that miR‐132 may be involved in the regulation of the pro‐epileptogenic TGF‐β pathway in human astrocytes.

### 3.4 miR‐132 overexpression downregulates IL‐1β‐induced factors

**Figure 5:**
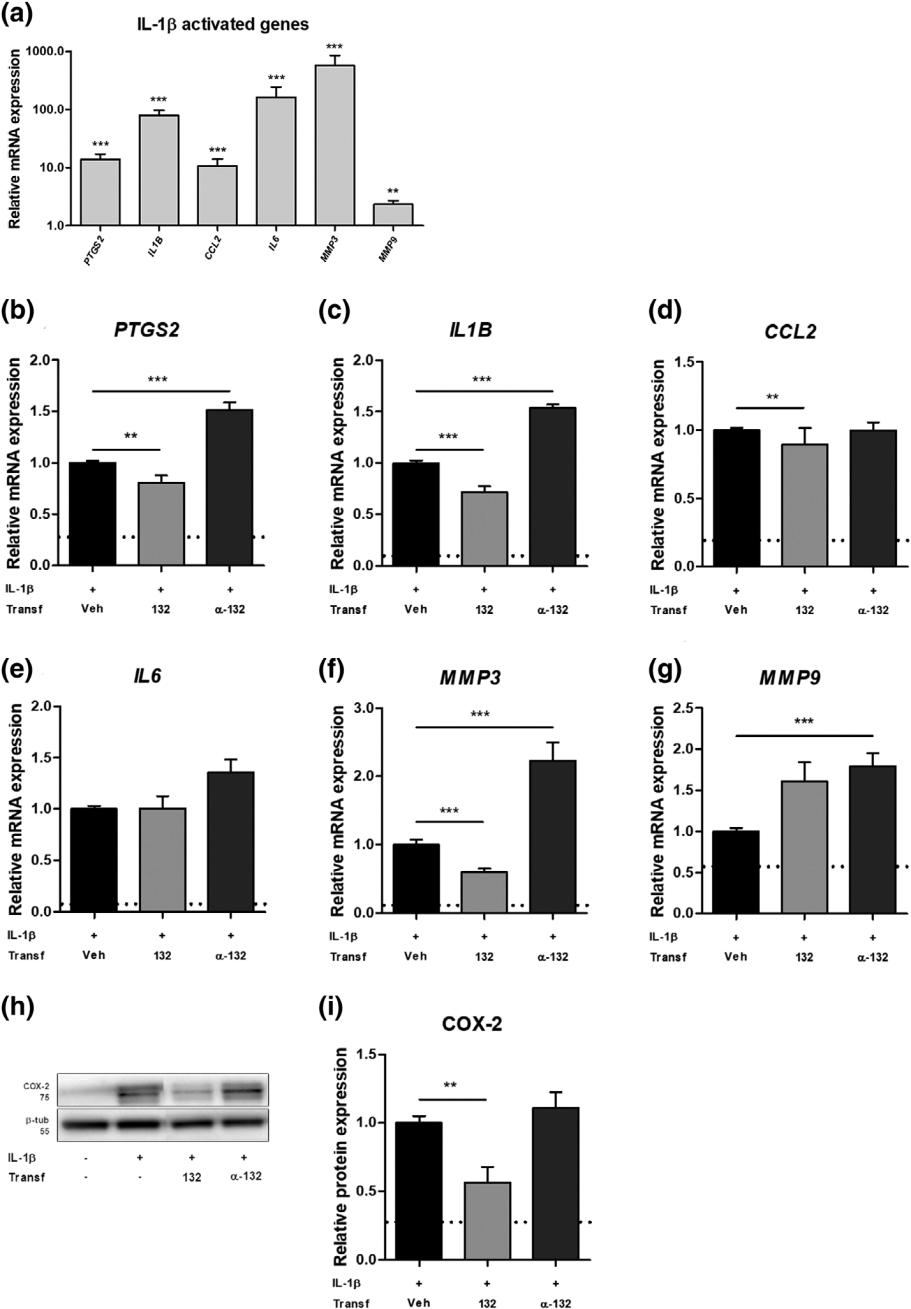
miR‐132 overexpression downregulates IL‐1β‐induced factors. The stimulation of primary human fetal astrocytes with IL‐1β resulted in increased expression of *PTGS2*, *IL1B*, *CCL2*, *IL6*, *MMP3* (all *p* < .001) and *MMP9* (*p* < .01) (a); miR‐132 transfection attenuated the increased expression of *PTGS2* (b, *p* < .01), *IL1B* (c, *p* < .001), *CCL2* (d, *p* < .01) and *MMP3* (f, *p* < .001), whereas inhibition of miR‐132 further increased the expression of *PTGS2* (b, *p* < .001), *IL1B* (c, *p* < .001), *MMP3* (f, *p* < .001) and *MMP9* (g, *p* < .001); no modulation of expression was found for *IL6* (e); semi‐quantitative analysis of the western blot showed that the expression of COX‐2 protein was also decreased (*p* < .01) after miR‐132 transfection (h, i); the data were normalized to the stimulated control condition and the dotted line indicates non‐stimulated control; veh, vehicle (Lipofectamine 2000); α‐132 indicates antagomir; *n* = 5 for RT‐qPCR, *n* = 3 for western blot, Mann–Whitney *U* test, *p < .05, **p < .01, ***p < .001

Next, we investigated the potential role of miR‐132 in astrocytes after stimulation of cells with IL‐1β, another pro‐epileptogenic pathway associated with neuroinflammation. As expected, the expression of a number of inflammatory genes was more than two‐fold higher after IL‐1β stimulation: prostaglandin‐endoperoxide synthase 2 (*PTGS2*), *IL1B*, C‐C Motif Chemokine Ligand 2 (*CCL2*), *IL6* (all *p* < .001), as well as *MMP3* (*p* < .001) and *MMP9* (*p* < .01; Figure 5a). The expression of miR‐132 did not change following IL‐1β treatment (Figure S1b). The expression of the miR‐132 predicted target gene *PTGS2* was 20% lower (*p* < .01) after miR‐132 overexpression and 50% higher (*p* < .001) after miR‐132 inhibition (Figure 5b). The same pattern was found for *IL1B* (Figure 5c). *CCL2* gene expression was also lower (*p* < .01) after miR‐132 overexpression (Figure 5d). The expression of *IL6* was not modulated by miR‐132 (Figure 5e). The expression of *MMP3* was modulated in the same way as *PTGS2* and *IL1B*, with miR‐132 mimic decreasing *MMP3* by 40% (*p* < .001) and miR‐132 antagomir further increasing (*p* < .001) *MMP3* expression 2.2‐fold (Figure 5f). The expression of *MMP9* was also 1.8‐fold higher after miR‐132 inhibition (*p* < .001, Figure 5g), although a trend toward upregulation was also seen for miR‐132 overexpression. TargetScan prediction showed a predicted binding site for miR‐132 in the 3′UTR of *PTGS2* mRNA (Figure S1c); therefore, we performed a western blot analysis, which confirmed that COX‐2 protein expression was also 40% lower after miR‐132 overexpression in astrocytes (*p* < .01; Figure 5h,i), confirming mRNA data. Thus, miR‐132 may act as a negative regulator of pro‐epileptogenic gene expression in human astrocytes during inflammatory conditions.

## 4 DISCUSSION

We investigated the expression of miR‐132 in the epileptogenic rat and human hippocampus as well as the effects of miR‐132 modulation in human cultured astrocytes after stimulation with epilepsy‐associated cytokines TGF‐β1 and IL‐1β. We showed a persistently increased expression of miR‐132 in the rat and human epileptogenic hippocampus, particularly in glial cells. Furthermore, we showed that miR‐132 can negatively regulate the expression of pro‐epileptogenic factors in cultured human astrocytes. These findings will be further discussed in more detail in the following paragraphs.

### 4.1 miR‐132 expression is increased in the rat and human epileptogenic hippocampus

miR‐132 is a cAMP‐response element binding protein (CREB)‐regulated miRNA, enriched in neurons (Jovicic et al., 2013), and involved in the regulation of neuronal morphogenesis (Vo et al., 2005) and dendritic spine density (Hansen, Sakamoto, Wayman, Impey, & Obrietan, 2010; Wayman et al., 2008), which implicates miR‐132 in the regulation of neuronal plasticity, memory, and learning (Aten et al., 2016). The expression of miR‐132 is dysregulated in a number of neurological pathologies (Soreq & Wolf, 2011) and increased miR‐132 expression was also found in resected brain tissue of children with TLE (Peng et al., 2013; Ren, Zhu, & Li, 2016). Here, we report that expression of miR‐132 is increased in the hippocampus of adult TLE‐HS patients. Moreover, miR‐132 was found to be increased in the epileptogenic DG of rats already at the acute stage of electrically‐induced epileptogenesis, which confirmed our previous microarray findings (Gorter et al., 2014), as well as reports from other large‐scale gene expression studies (Bot, Debski, & Lukasiuk, 2013; Kretschmann et al., 2015). The increased miR‐132 expression in the rodent hippocampus has also been shown following administration of other pro‐epileptogenic stimuli including pilocarpine (Nudelman et al., 2010; Peng et al., 2013) and kainic acid (Jimenez‐Mateos et al., 2011), and a meta‐analysis of differentially expressed miRNAs across various animal TLE models identified miR‐132 as one of the most commonly up‐regulated miRNAs during the acute, latent and chronic stages of epileptogenesis (Korotkov et al., 2017), highlighting its potential involvement in the pathophysiology of epilepsy. Our histological analysis showed that miR‐132 expression in the rat hippocampus was increased in previously quiescent glial cells, including astrocytes and microglia. However, previous studies both in healthy and pathological tissue have focused exclusively on neuronal miR‐132 expression and little is known about potential miR‐132 expression and function in glial cells. Therefore, we further investigated whether this phenomenon could also be observed in human TLE‐HS tissue.

### 4.2 miR‐132 is expressed in glial cells in human TLE‐HS

Increased expression of miR‐132 was detected in astrocytes and microglia within the hippocampus of TLE patients. At the same time, the expression of miR‐132 in the principal neuronal layers of the hippocampus was reduced, consistent with the phenomena of granule cell dispersion in the DG and neuronal damage in the CA1. We observed moderate‐to‐severe astrogliosis in the hippocampus of TLE patients. However, it has been shown that various subtypes of reactive astrocytes may exist based on their gene expression profiles (Zamanian et al., 2012). The pro‐inflammatory astrocyte response may lead to a loss of normal physiological functions by astrocytes, which become a source and mediators of chronic neuroinflammation (Aronica, Ravizza, Zurolo, & Vezzani, 2012; Liddelow & Barres, 2017). On the other hand, reactive astrocytes may participate in glial scar formation and produce neurotrophins and thrombospondins, aiding synapse formation and repair (Liddelow & Barres, 2017; Sofroniew, 2015), which may lead to epilepsy. Therefore, we investigated whether miR‐132 expression was co‐localized with certain markers of reactive glial cells.

miR‐132 expression in the hippocampus of patients with TLE was observed in association with various astrocyte markers, including GFAP, EAAT1, GS, and vimentin, a marker of reactive astrocytes. Additionally, miR‐132 expressing STAT3^+^ cells were detected in the areas of severe gliosis and glial scar in the CA1 region. The JAK‐STAT3 pathway regulates cell growth, proliferation, differentiation, and response to injury in astrocytes (Ceyzeriat, Abjean, Carrillo‐de Sauvage, Ben Haim, & Escartin, 2016) and has been proposed as a specific marker of glial scar‐forming reactive astrocytes (Anderson et al., 2016). Microglia expressed miR‐132 as well, since we detected miR‐132 in Iba1^+^ cells and reactive HLA‐DR/DP/DQ^+^ microglia. Interestingly, the expression of miR‐132 could be induced in microglia under oxygen and glucose deprivation conditions in vitro (Kong et al., 2014). Finally, the co‐localization of miR‐132 and the endothelial cell marker CD34 was found to be associated with the brain blood vessels. Notably, the increased expression of miR‐132 in the endothelium of human tumors and hemangiomas has been shown to promote neovascularization (Anand et al., 2010). It is worth noting that neurons—the primary source of miR‐132 in the brain—may be able to secrete miR‐132‐carrying exosomes, which could influence vascular integrity (Xu et al., 2017). This suggests that miR‐132 in non‐neuronal cells could have an exogenous origin, however we observed a mostly perinuclear miR‐132 hybridization signal in astrocyte somas and no hybridization signal in astrocytic processes. Thus, miR‐132 is expressed in a wide range of cell types in the rat and human TLE hippocampus and could be associated with diverse functions, extending beyond the known functions in neurons.

### 4.3 miR‐132 acts as a negative regulator of pro‐epileptogenic factors induced by TGF‐β1 and IL‐1β

In order to study the potential functions of miR‐132 in astrocytes, we performed experiments in primary human astrocyte cultures after stimulation with cytokines associated with epileptogenesis. The analysis of predicted target genes identified the TGF‐β pathway as one of the most enriched pathways associated with miR‐132. The activation of the TGF‐β pathway by TGF‐β1 in astrocytes not only increased the transcription of the genes directly involved in the pathway, such as *TGFB1*, *TGFB2*, and *TGFBR1*, but also led to an increase in ECM‐related genes *THBS1* and matrix metalloproteinases *MMP2* and *MMP14*. Interestingly, the stimulation with TGF‐β1 also led to an increased expression of miR‐132. Previously, miR‐132 induction has been shown in human astrocytic cell line by a pro‐inflammatory myeloid‐related protein (MRP) 8 (Kong et al., 2015) and in primary rat astrocytic cultures by basic fibroblast growth factor (bFGF; Numakawa et al., 2011), confirming the inducible nature of miR‐132 expression in glial cells. We investigated the effects of miR‐132 overexpression on the expression of its predicted targets within the TGF‐β pathway and found that the expression of *TGFB2* as well as TGF‐β2 protein could be attenuated by miR‐132. TGF‐β signaling can have pro‐epileptogenic functions after a brain insult. The disruption of the BBB and extravasation of blood albumin has been shown to activate TGF‐β signaling in astrocytes and contribute to epileptogenesis (Cacheaux et al., 2009; Ivens et al., 2007), potentially through induction of synaptogenesis (Weissberg et al., 2015). Moreover, the treatment of rats with an angiotensin II Type 1 (AT1) receptor antagonist losartan can block TGF‐β signaling and prevent the development of recurrent spontaneous seizures and seizure severity following the BBB breakdown (Bar‐Klein et al., 2014). In this regard, the modulation of TGF‐β2 by miR‐132 could be beneficial for prevention of epileptogenesis. However, miR‐132 overexpression lead to an increased expression of *TGFBR2*, which is also increased upon suppression of miR‐211, and associated with a nonconvulsive electrographical seizures and increased convulsive seizure susceptibility in mice (Bekenstein et al., 2017). Moreover, TGF‐β signaling in astrocytes has been also shown to be necessary for limiting neuroinflammation in a mouse experimental stroke model (Cekanaviciute et al., 2014), whereas treatment of rat primary astrocytes with TGF‐β1 could completely reverse the pro‐inflammatory astrocytic phenotype caused by IL‐1α, tumor necrosis factor alpha (TNF‐α) and complement component 1q (C1q) stimulation (Liddelow et al., 2017).

We next assessed the effect of miR‐132 on pro‐epileptogenic factors associated with a pro‐inflammatory cytokine IL‐1β. Among the predicted targets of miR‐132 are *PTGS2* and its product COX‐2, a key regulator of inflammation mediated by IL‐1β. We found that both *PTGS2* and COX‐2 were decreased following miR‐132 overexpression. COX‐2 is normally expressed in neurons, but it has also been found in astrocytes in human TLE (Desjardins et al., 2003) and in an animal TLE model (Holtman et al., 2009). Moreover, the expression of other pro‐inflammatory genes associated with epilepsy, such as *IL1B* and *CCL2*, was decreased by miR‐132 overexpression in astrocytes. This suggests an anti‐inflammatory mode of action by miR‐132. This is in accordance with previous studies, since miR‐132 has been shown to have anti‐inflammatory action in the brain via targeting acetylcholinesterase (Shaked et al., 2009). Moreover, inhibition of miR‐132 in a human astrocytic cell line under stimulation with MRP8 promoted the expression of pro‐inflammatory genes *IL1B*, *IL6*, and *TNF*, whereas upregulation of miR‐132 suppressed the transcription of these genes (Kong et al., 2015).

In addition, both TGF‐β1 and IL‐1β are responsible for transcription of genes, which take part in ECM remodeling. We found that TGF‐β1 stimulation increased the constitutively expressed *MMP2* and *MMP14*, whereas the inducible *MMP3* and *MMP9* could be increased by IL‐1β stimulation in human astrocytes. MMPs are the major enzymes responsible for the remodeling of the ECM (Rempe et al., 2016) and we previously demonstrated the dynamic expression of *Mmp2*, *Mmp3*, *Mmp9*, and *Mmp14* during epileptogenesis in the rat hippocampus (Gorter et al., 2007; Korotkov et al., 2018). miR‐132 overexpression negatively regulated *MMP3* and inhibition of miR‐132 led to a further increase in *MMP9*, a metallopeptidase implicated in epileptogenesis (Wilczynski et al., 2008). In summary, miR‐132 may act as a negative regulator of various pro‐epileptogenic factors induced by TGF‐β1 and IL‐1β stimulation in human astrocytes.

### 4.4 Therapeutic potential of miR‐132

Due to the suppression of pro‐epileptogenic factors (including pro‐inflammatory and ECM factors), it would be interesting to investigate whether miR‐132 could be used as a novel strategy to inhibit epileptogenesis. It has been previously shown that intracerebroventricular injection of an antagomir of miR‐132 protected against hippocampal CA3 neuronal death 24 hr after SE induced via intra‐amygdala injection of kainic acid (Jimenez‐Mateos et al., 2011). This suggests that miR‐132‐overexpressing therapy can aggravate neuronal damage, however, the authors did not investigate the later time points after SE, at which brain inflammation and ECM alterations are much more prominent. It is interesting to note that inhibition of miR‐132 in astrocytes further augmented the expression of inflammatory genes in our study, which may result in exacerbation of astrocyte‐mediated inflammatory response in case of using miR‐132 inhibitors in vivo. Therefore, this needs to be studied and the optimal time window for miR‐132 therapy has to be determined.

## 5 CONCLUSIONS

Our experiments showed that miR‐132 is persistently increased in glial cells in the human and rat epileptogenic brain. Since miR‐132 can act as a negative regulator of epilepsy‐associated factors in cultured human astrocytes, the therapeutic potential of miR‐132, specifically in astrocytes, needs to be further studied in in vivo experiments.

## CONFLICT OF INTEREST

None of the authors has any conflict of interest to disclose.

## AUTHORS' CONTRIBUTIONS

A.K., D.W.M.B., L.B., B.P., J.v.S., E.A.v.V., and E.A. conceived and designed the analysis. J.v.S. and J.J.A. helped with experimental work and methodology. J.C.B. and S.I. provided human patient tissue. A.K. and D.W.M.B. analyzed the data. J.G., E.A.v.V., and E.A. contributed to the data interpretation and writing of the manuscript. All authors read, revised, and approved the final manuscript.

## Supporting information


**Table S1** The list of human primers and oligonucleotide probe. f ‐ forward, r ‐ reverse; anti‐miR‐132‐3p oligonucleotide probe for in situ hybridization had the following modifications: * ‐ locked nucleic acid (LNA) modification; m ‐ 2‐o‐methyl modification; DIG ‐ digoxygenin label

**Figure S1**. (a) — Targetscan prediction of the miR‐132 binding site within the human *TGFB2* 3’ UTR; (b) — Taqman RT‐qPCR analysis did not show differences in miR‐132 expression following IL‐1ß stimulation in human primary fetal astrocytes; (c) — Targetscan prediction of the miR‐132 binding site within the human *PTGS2* 3’ UTR; Mann–Whitney *U* test
