# Supplementary material for: microRNA‐132 is overexpressed in glia in temporal lobe epilepsy and reduces the expression of pro‐epileptogenic factors in human cultured astrocytes
Source: Glia. 2019 Aug 13;68(1):60–75. doi: 10.1002/glia.23700 (PMC6899748; doi:10.1002/glia.23700)
Supplement: Supplementary file 1 — Table S1 The list of human primers and oligonucleotide probe. f ‐ forward, r ‐ reverse; anti‐miR‐132‐3p oligonucleotide probe for in situ hybridization had the following modifications: * ‐ locked nucleic acid (LNA) modification; m ‐ 2‐o‐methyl modification; DIG ‐ digoxygenin label [file GLIA-68-60-s002.docx]

| **Human primers for RT-qPCR** | | | |
| --- | --- | --- | --- |
| Gene | Full gene name | f/r | Sequence |
| *TGFB1* | Transforming growth factor beta 1 | f | GTGGAAACCCACAACGAAAT |
|  |  | r | CGGAGCTCTGATGTGTTGAA |
| *TGFB2* | Transforming growth factor beta 2 | f | GAGAGCGCAAGTGAAAGAGG |
|  |  | r | TCCCTAGACCGTCAGGCTAA |
| *TGFBR1* | Transforming growth factor beta receptor 1 | f | AAGAACGTTCGTGGTTCCGT |
|  |  | r | CACCAACCAGAGCTGAGTCC |
| *TGFBR2* | Transforming growth factor beta receptor 2 | f | CCACCGCACGTTCAGAAGTC |
|  |  | r | GTCCTATTACAGCTGGGGCA |
| *SMAD2* | Mothers against decapentaplegic homolog 2 | f | AAAGGGTGGGGAGCAGAATA |
|  |  | r | GAAGTTCAATCCAGCAAGGAGT |
| *THBS1* | Thrombospondin 1 | f | GCGTCAATGACAATTTCCAG |
|  |  | r | TCAAGGGTGAGGAGGACACT |
| *PTGS2* | Prostaglandin-endoperoxide synthase 2 | f | GAATGGGGTGATGAGCAGTT |
|  |  | r | GCCACTCAAGTGTTGCACAT |
| *IL1B* | Interleukin 1 beta | f | GCATCCAGCTACGAATCTCC |
|  |  | r | GAACCAGCATCTTCCTCAGC |
| *IL6* | Interleukin 6 | f | CTCAGCCCTGAGAAAGGAGA |
|  |  | r | TTTCAGCCATCTTTGGAAGG |
| *CCL2* | Chemokine (C-C motif) ligand 2 | f | CTGCTCATAGCAGCCACCTT |
|  |  | r | GCACTGAGATCTTCCTATTGGTG |
| *MMP2* | Matrix metallopeptidase 2 | f | ATAACCTGGATGCCGTCGT |
|  |  | r | AGGCACCCTTGAAGAAGTAGC |
| *MMP3* | Matrix metallopeptidase 3 | f | CTCCAACCGTGAGGAAAATC |
|  |  | r | CATGGAATTTCTCTTCTCATCAAA |
| *MMP9* | Matrix metallopeptidase 9 | f | GAACCAATCTCACCGACAGG |
|  |  | r | GCCACCCGAGTGTAACCATA |
| *MMP14* | Matrix metallopeptidase 14 | f | GCCTTGGACTGTCAGGAATG |
|  |  | r | AGGGGTCACTGGAATGCTC |
| *EF1a* | Elongation factor 1 alpha | f | ATCCACCTTTGGGTCGCTTT |
|  |  | r | CCGCAACTGTCTGTCTCATATCAC |
| *C1orf43* | Chromosome 1 open reading frame 43 | f | GATTTCCCTGGGTTTCCAGT |
|  |  | r | ATTCGACTCTCCAGGGTTCA |

**anti-hsa-miR-132-3p LNA probe used for *in situ* hybridization:**

5'DIG-*CmGmA*CmCmA*TmGmG*CmUmG*TmAmG*AmCmU*GmUmU*A-DIG3'

**Supplementary table S1.** The list of oligonucleotides. Human primers were designed for Homo sapiens, MMP – matrix metalloproteinase, EF1a - elongation factor 1α, C1orf43 - chromosome 1 open reading frame 43; f - forward, r - reverse; anti-miR-132-3p oligonucleotide probe for in situ hybridization had the following modifications: * - locked nucleic acid (LNA) modification; m - 2-o-methyl modification; DIG - digoxygenin label
