# Supplementary figures and images for: microRNA‐132 is overexpressed in glia in temporal lobe epilepsy and reduces the expression of pro‐epileptogenic factors in human cultured astrocytes
Source: Glia. 2019 Aug 13;68(1):60–75. doi: 10.1002/glia.23700 (PMC6899748; doi:10.1002/glia.23700)

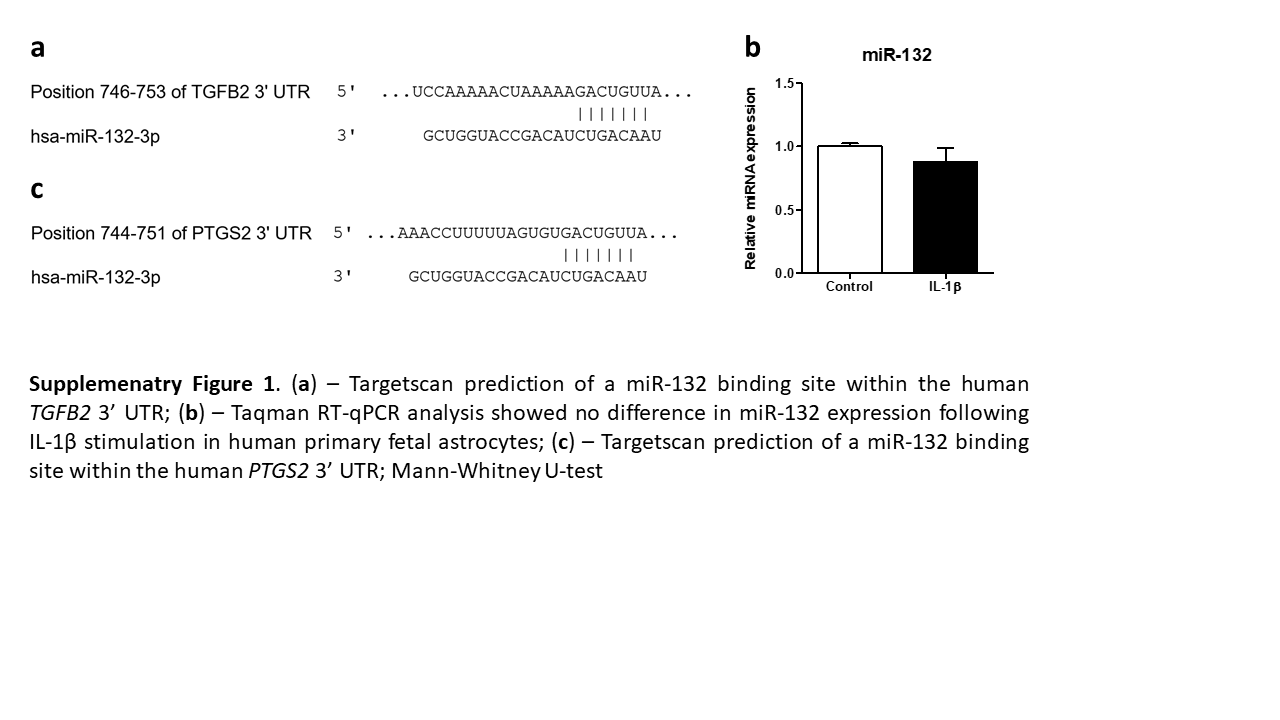

Supplement: Supplementary file 2 — Figure S1. (a) — Targetscan prediction of the miR‐132 binding site within the human TGFB2 3’ UTR; (b) — Taqman RT‐qPCR analysis did not show differences in miR‐132 expression following IL‐1ß stimulation in human primary fetal astrocytes; (c) — Targetscan prediction of the miR‐132 binding site within the human PTGS2 3’ UTR; Mann–Whitney U test [file GLIA-68-60-s001.tif]
